# Retraction: Interaction of Wnt pathway related variants with type 2 diabetes in a Chinese Han population

**DOI:** 10.7717/peerj.1304/retraction

**Published:** 2022-11-18

**Authors:** PeerJ Editorial Office

Retraction: Zhou JB, Yang JK, Zhang BH, Lu J. 2015. Interaction of Wnt pathway related variants with type 2 diabetes in a Chinese Han population. PeerJ 3:e1304 https://doi.org/10.7717/peerj.1304

Following a complaint by Dr Jin-Kui Yang (the 2nd named author) it transpired that this submission was made without the knowledge or approval of either Dr Jin-Kui Yang or Dr Jing Lu (the 4th named author). As a result, Dr Jian-Bo Zhou (the 1st named author) requested Retraction of the article.

PeerJ Editorial Office. 2022. Retraction: Interaction of Wnt pathway related variants with type 2 diabetes in a Chinese Han population. PeerJ 3:e1304/retraction https://doi.org/10.7717/peerj-e1304/retraction.

